# Differential effect of Platelet Endothelial Cell Adhesion Molecule-1 (PECAM-1) on leukocyte infiltration during contact hypersensitivity responses

**DOI:** 10.7717/peerj.3555

**Published:** 2017-07-10

**Authors:** Merideth Early, William G. Schroeder, Ranajana Unnithan, John M. Gilchrist, William A. Muller, Alan Schenkel

**Affiliations:** 1 Department of Microbiology, Immunology, and Pathology, Colorado State University, Fort Collins, CO, United States of America; 2 Department of Pediatrics, University of Colorado Health Sciences Center, Aurora, CO, United States of America; 3 Department of Physiology, University of California, San Francisco, United States of America; 4 Department of Pathology, Northwestern University, Chicago, IL, United States of America

**Keywords:** Platelet Endothelial Cell Adhesion Molecule-1, Leukocyte, Contact Hypersensitivity, Emigration, Monocytes, Granulocytes, γδT lymphocytes, CD4+ T lymphocytes, Natural Killer Lymphocytes

## Abstract

**Background:**

2′–4′ Dinitrofluorobenzene (DNFB) induced contact hypersensitivity is an established model of contact sensitivity and leukocyte migration. Platelet Endothelial Cell Adhesion Molecule-1 (PECAM-1) deficient mice were used to examine the role of PECAM-1 in the migration capacity of several different leukocyte populations after primary and secondary application.

**Results:**

γδ T lymphocytes, granulocytes, and Natural Killer cells were most affected by PECAM-1 deficiency at the primary site of application. γδ T lymphocytes, granulocytes, DX5+ Natural Killer cells, and, interestingly, effector CD4+ T lymphocytes were most affected by the loss of PECAM-1 at the secondary site of application.

**Conclusions:**

PECAM-1 is used by many leukocyte populations for migration, but there are clearly differential effects on the usage by each subset. Further, the overall kinetics of each population varied between primary and secondary application, with large relative increases in γδ T lymphocytes during the secondary response.

## Introduction

2′–4′ Dinitrofluorobenzene (DNFB) induced contact hypersensitivity is an established model of inflammation. Haptenylation of proteins by DNFB induces a type IV delayed hypersensitivity (Asherson & Ptak, 1968). One of the most important recent advances in the field of Natural Killer (NK) lymphocyte immunology was the discovery that not only do NK cells contribute to this response, but also that they have a memory-recall response which resides in a liver CXCR6+ population (O’Leary et al., 2006; Paust et al., 2010).

Platelet Endothelial Cell Adhesion Molecule-1 (PECAM-1, CD31) is expressed on platelets, endothelial cells and all leukocytes, while it is shed on activated B and T lymphocytes (Fornasa et al., 2010; Jackson et al., 2000; Muller, 2001). PECAM-1 is expressed at endothelial cell junctions and PECAM-1 homophilic interactions are important as leukocytes undergo extravasation into inflamed tissues (Schenkel, Chew & Muller, 2004).

The role of PECAM-1 had not previously been studied in the context of DNFB-induced contact hypersensitivity nor on γδ T and NK lymphocytes. In this study, we tested both the primary and secondary responses of γδ T and NK lymphocytes in wild type and PECAM-1 deficient mice. There are relatively few papers studying the effects of DNFB on γδ T cells (Askenase et al., 2011; Nielsen et al., 2014; Shi et al., 2012) but this population plays an important role in skin contact hypersensitivity reactions.

## Materials & Methods

### Ethics statement

Colorado State University Institutional Animal Care and Compliance committee approved all protocols related to this project (Approval ID 05-153).

#### Animals

Age (2–4 months) and gender matched male and female wild type and PECAM-1 deficient mice in the FVB/n strain were used for all experiments. Due to the spontaneous pulmonary fibrosis in the PECAM-1 deficient mice in this strain (Schenkel et al., 2006), any animals showing disease were excluded after screening by blood oxygen saturation (Early et al., 2009). For all experiments, 5–6 wild type or PECAM-1 deficient mice were used in each group. Each experiment (primary or secondary exposure) was repeated 3 times.

#### Primary DNFB exposure

25 ul of 0.5% DNFB in olive oil was applied on ∼1 cm^2^ unshaved flank skin by directly pipetting onto the surface. We used unshaven skin because sometimes shaving caused skin irritation. Skin was harvested 48 h later for histologic and flow cytometric analysis. For flow cytometry, skin was trimmed to 3 cm × 5 cm sections before digestion and cell counts.

#### Secondary DNFB exposure

Five days after primary exposure, 10 ul of 0.15% DNFB in olive oil was applied to one ear; the opposite ear was also treated with olive oil to account for grooming of the skin. Forty-eight hours later, ears were harvested and fixed in 10% buffered formalin for histology or digested in collagenase for flow cytometry.

#### Flow cytometry

Ears or skin were incubated in RPMI 1640 (Life Technologies, Gaithersburg, MD) containing collagenase IV (0.7 mg/ml) for 30 min, then RPMI containing 10% fetal bovine serum was added to stop digestion. The entire mixture was filtered through 40 um filters to collect cells by centrifugation. Cold phosphate buffered saline was used for all further staining steps. Cells were pre-blocked in 1% normal rat serum and Fc-blocking antibodies before staining with the following antibodies or isotype-matched color controls.

All antibodies (Table 1) and isotype color controls were purchased from eBioscience (San Diego, CA) except for CD206 (Biolegend, San Diego, CA, USA). Clones indicated in parentheses. Flow cytometry was performed on a Dako Cyan (Carpinteria, CA, USA) and analyzed using FlowJo (Ashland, OR, USA) and JMP statistical (SAS, Cary, NC, USA) software.

**Table 1:** Flow cytometry reagents used.

Color	CD4+ T	CD8+ T	CD19+ B	NK/γδ	Myeloid cells
APC CY7	CD3 (145-2C11)	CD3	CD3 (-)	CD3	
PE CY5					
Pacific blue	CD4 (L3-T4)	CD8 (53-6.7)	CD19 (6D5)	CD8	CD11c (N418)
FITC	CD44 (IM7)	CD44	CD5 (53-7.3)	pan-γδ T cell (GL3)	CD11b (M1/70)
APC	CD62L (MEL-14)	CD62L	CD62L		GR1 (RB6-8C5)
PE Cy7				NKG2D (CX5)	CD206 (C068C2)
PE			IgD (11-26c)	DX5 (CD49b)	CD115 (AFS98)

Gating scheme: Live/dead exclusion was not used because some cells might be missed (dying neutrophils) and few dead cells were found outside of the typical debris or doublet gates. CD3+ CD4+ or CD3+ CD8+ T cells were analyzed for CD44 and CD62L markers of activation status. CD19+ CD3− B cells were analyzed for CD5 and IgD. NK cells were defined as CD3−, γδ TCR−, NKG2D+, DX5/CD49b+. CD3+ γδ TCR+ T cells were also defined by CD8 high or low. Neutrophils were defined by GR1+ CD11b+ CD11c^low∕neg^, CD115−. Dendritic cells were defined as CD11c+ CD11b^low^. Monocytes and macrophages were defined as CD11b+ CD11c+ CD115+ (Gonzalez-Juarrero et al., 2003; G Randolph, pers. comm., 2014).

#### Statistics

Samples from primary and secondary exposures to DNFB were done a minimum of three times with 4–6 mice in each group (PECAM-1 deficient mice and wild type mice) after testing for power. JMP statistical software (SAS, Cary, NC, USA) was used to analyze differences between groups using Tukey-Kramer Honestly Significant Different or One Way ANOVA tests.

## Results

**Figure 1:**
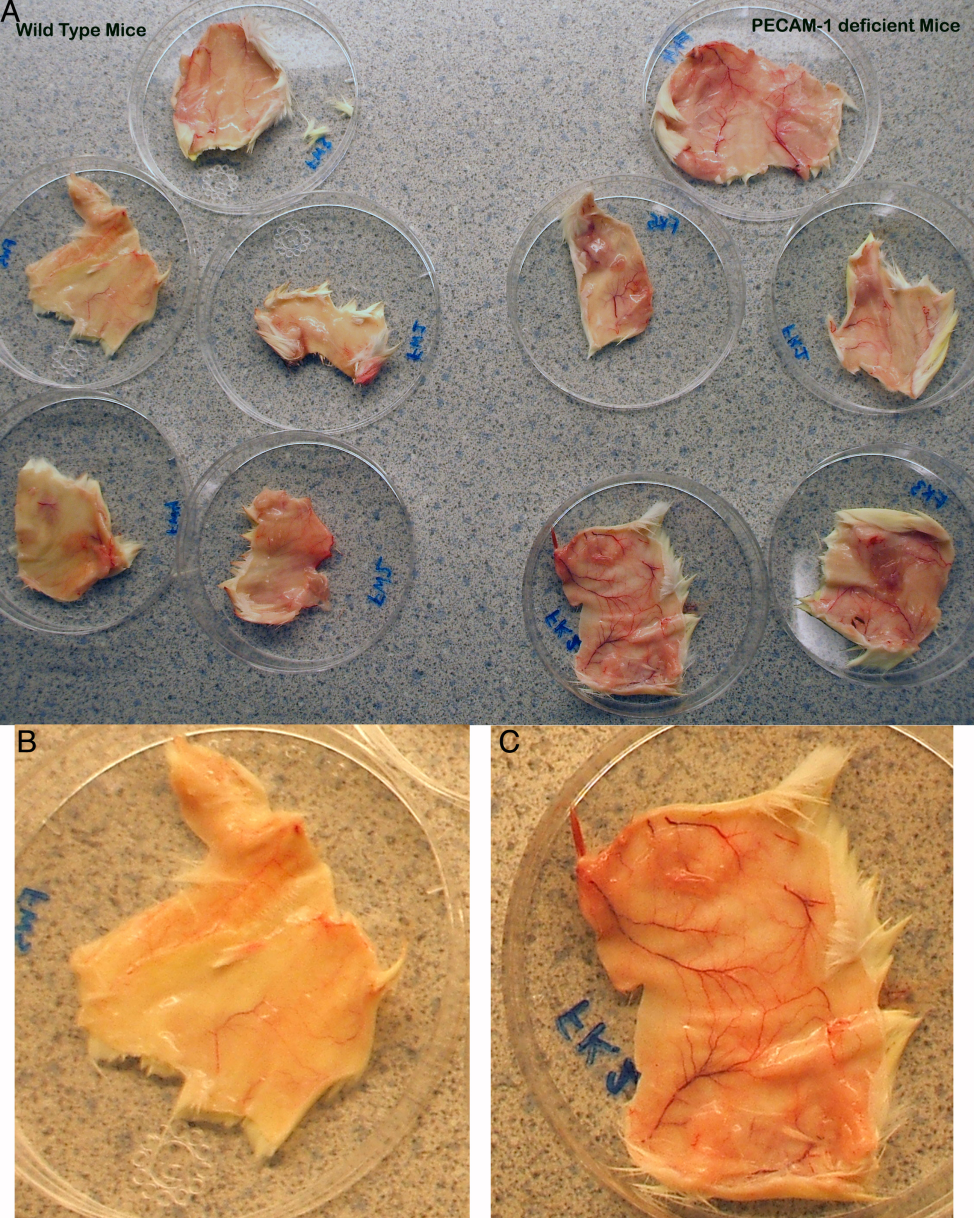
Gross anatomy of the skin at the flank site of primary DNFB application 28 h after application. The dilation of vasculature is much more prominent in the PECAM-1 deficient mice. (A) Overview of five wild type and five PECAM-1 deficient skin flanks after dissection before trimming to 3 cm × 5 cm size and collagenase digest. (B) Wild type, and (C) PECAM-1 deficient, show higher magnification examples.

The immune response to primary DNFB exposure in PECAM-1 deficient mice has not previously been reported. Upon dissection of the flank skin at the site of a single DNFB exposure, we found that the blood vessels were more dilated around the major vessels in PECAM-1 deficient mice than in wild type mice (Fig. 1).

**Figure 2:**
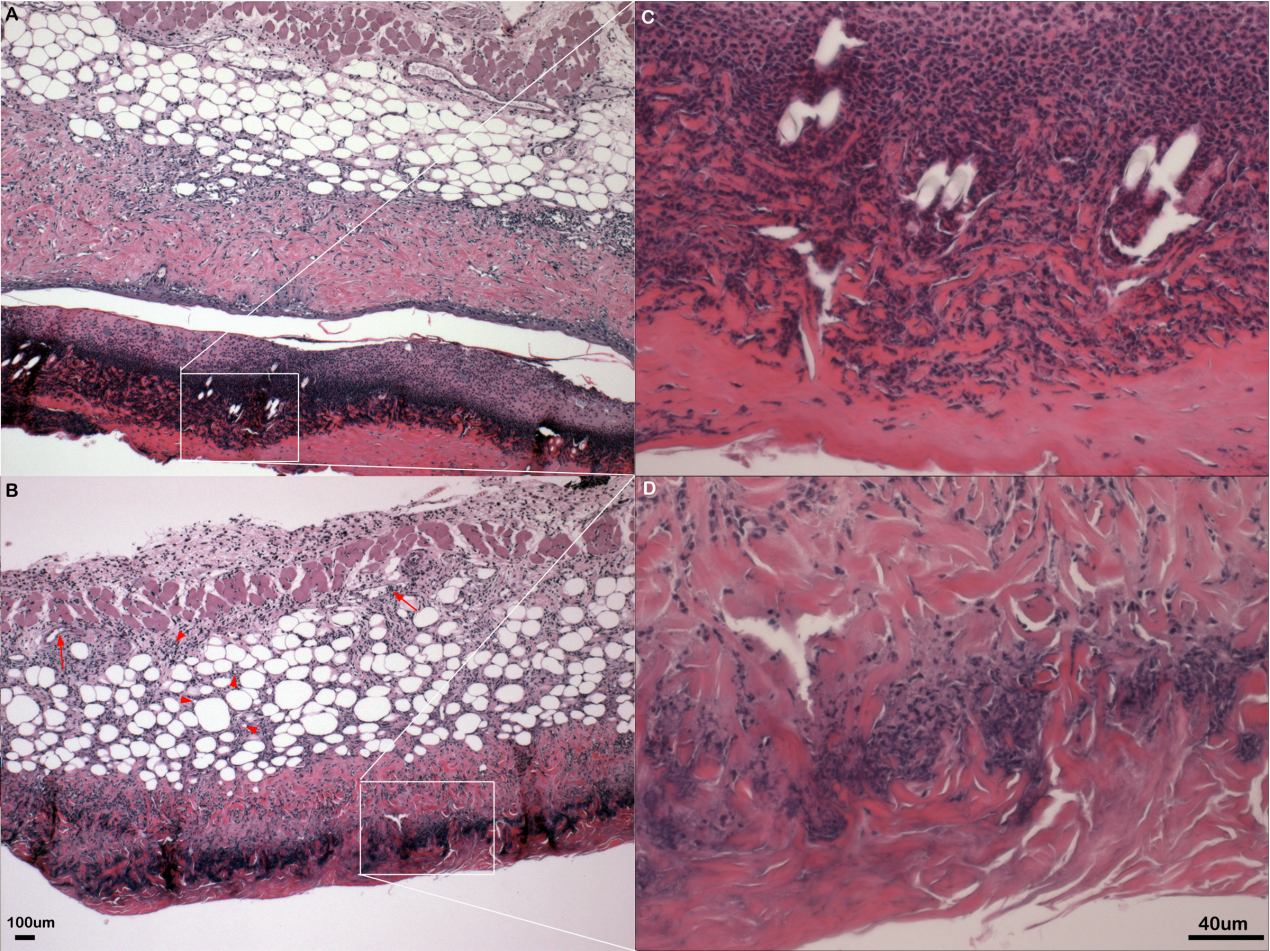
Inflammation in primary flank skin exposure to DNFB. Representative sections of inflammation in primary flank skin exposure to DNFB from wild type ((A) 2× magnification; (C) 20× magnification) and PECAM-1 deficient ((B) 2× magnification; (D) 20× magnification) mice.

**Figure 3:**
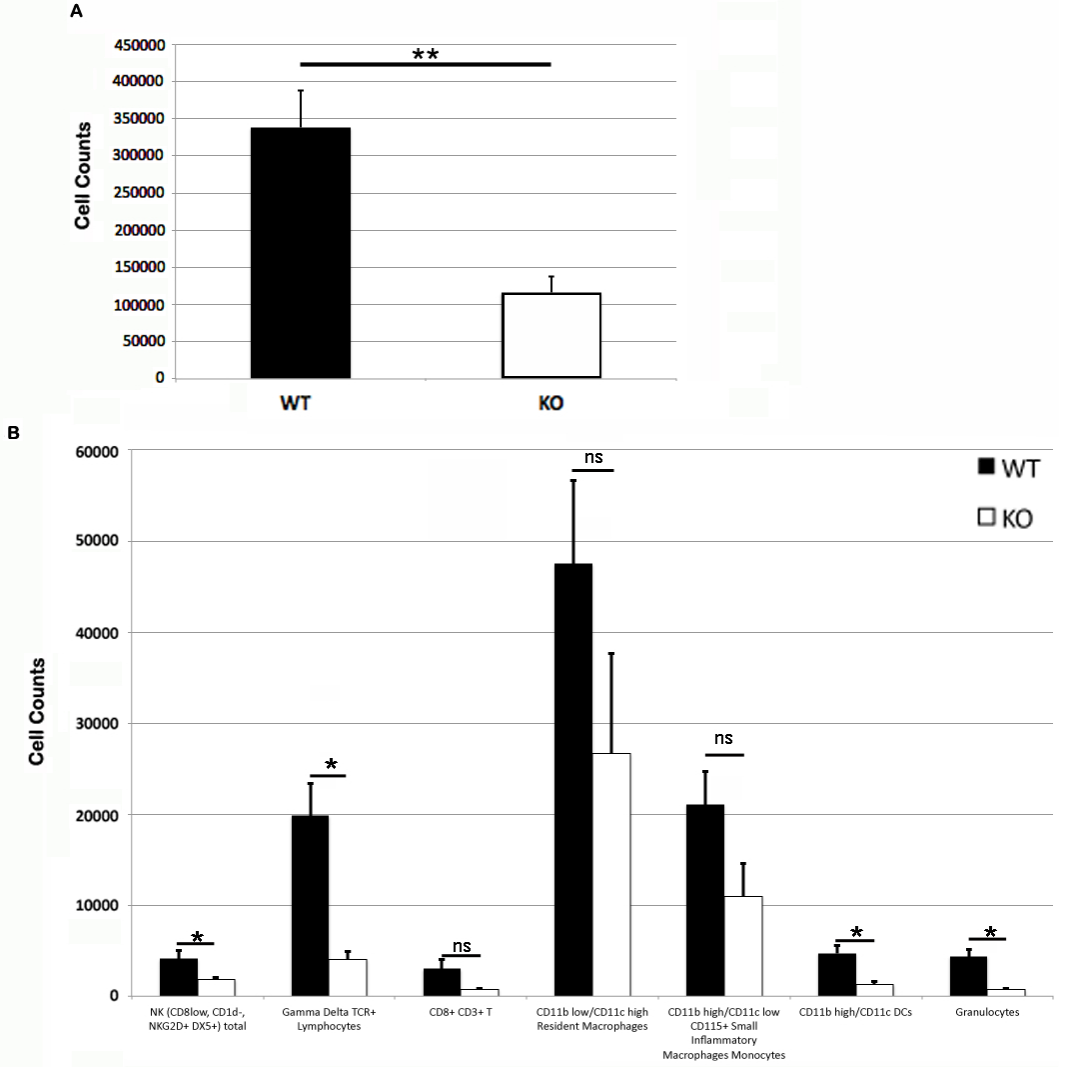
Leukocyte counts at primary DNFB exposure flank skin. Total (A) and leukocyte subset (B) cell counts from wild type and PECAM-1 deficient flank skin at the treatment sites, 48 h after DNFB application. ^∗^*p* < 0.05, ^∗∗^*p* < 0.01, Tukey Kramer HSD test. ns, not significant difference.

Somewhat paradoxically to the gross appearance, the flank skin layer had a larger inflammatory infiltrate of cells in wild type mice, primarily mononuclear cells with relatively few neutrophils (Figs. 2A and 2C) compared to PECAM-1 deficient mice (Figs. 2B and 2C). After collagenase digestion of flank skin tissue and collection of cells, we found significantly fewer total cells in PECAM-1 deficient mouse skin (Fig. 3A) despite using equal sized (3 cm × 5 cm) sections from both mice. Of the cells that were positive for the leukocyte panels we used, most leukocytes in the dermis were monocyte/macrophage phenotype, with differential effects in the two major populations CD11b^low^/CD11c^high^ “resident” macrophages not as affected as CD11b^high^/CD11c^low^ monocyte/small inflammatory macrophages (Fig. 3B). γδ TCR+ lymphocytes and CD11b^high^/CD11c^high^dendritic cells were significantly lower in PECAM-1 deficient mice. Using two markers, NKG2D and DX5, for Natural Killer cells, we found they were significantly lower in PECAM-1 deficient mice as well. CD8^bright^ CD3+ cells were found at low levels and not significantly lowered by PECAM deficiency although numbers were lower overall.

**Figure 4:**
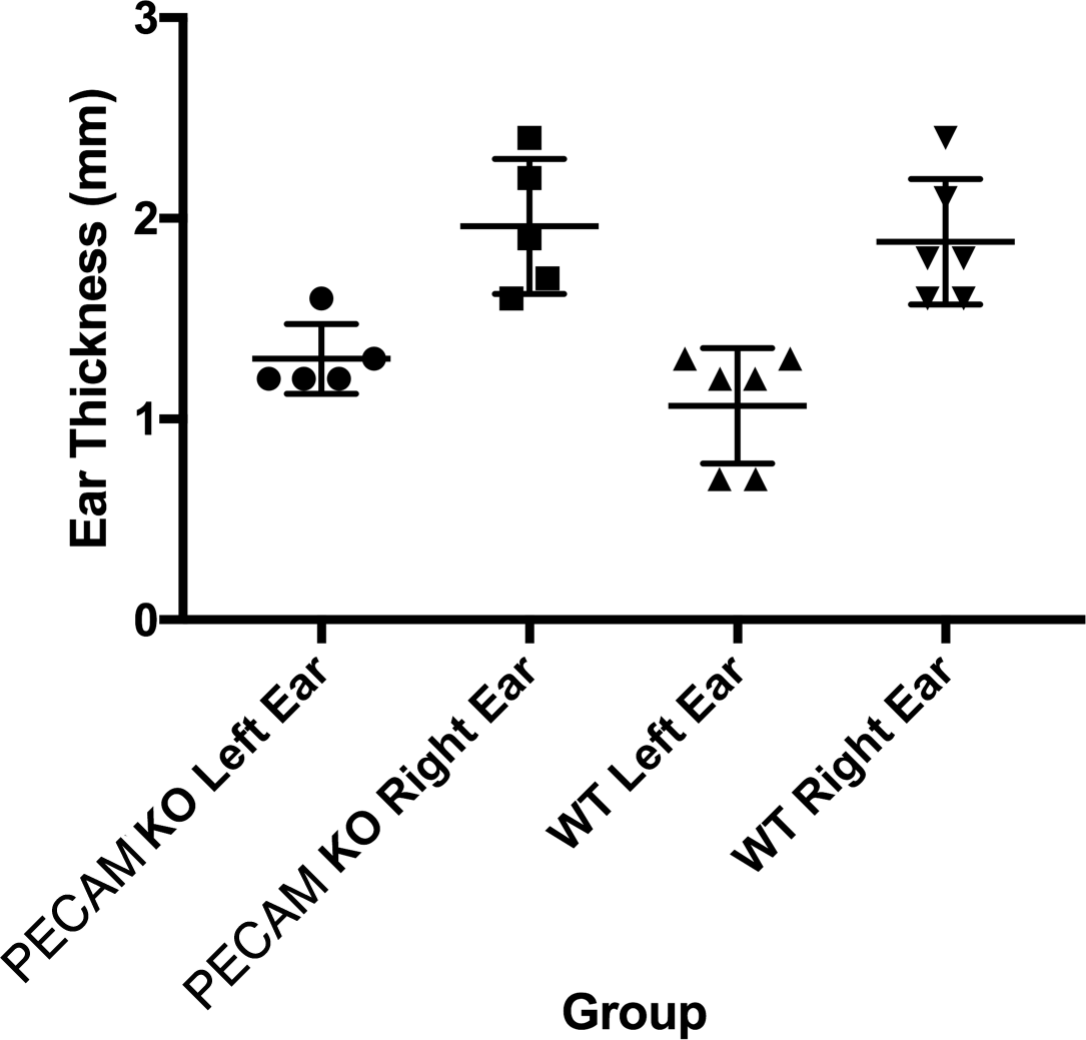
Ear swelling measurements. Right ear swelling by skin caliper measurement upon secondary exposure to DNFB in wild-type and PECAM-1 KO mice. Left ears were measured as controls for each animal.

**Figure 5:**
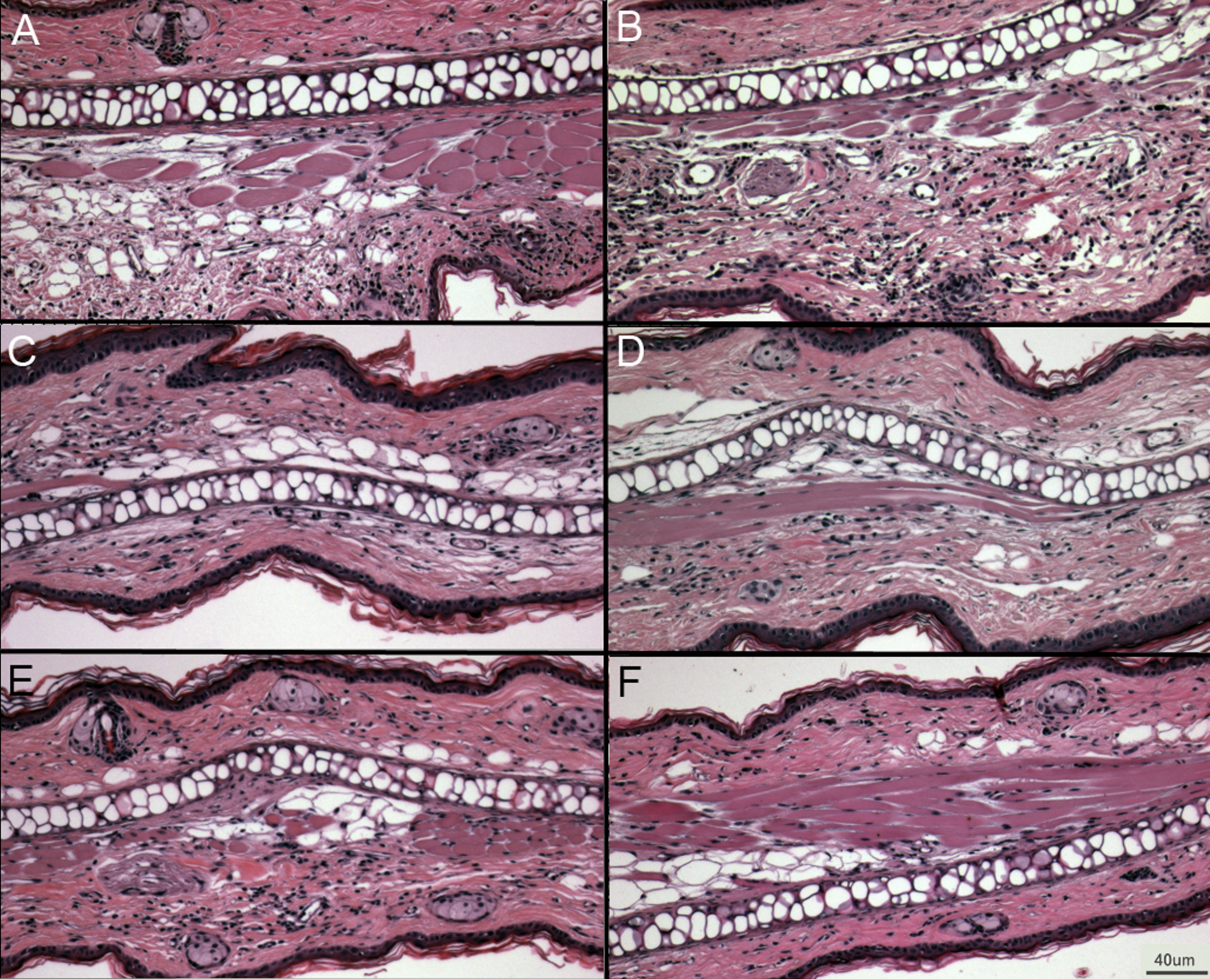
Representative ear sections after DNFB treatment. (A–B) DNFB treated wild type ears. (C–E) DNFB treated PECAM KO ears. (F) Untreated wild type controls and PECAM KO were equivalent in examination. 20× magnification.

**Figure 6:**
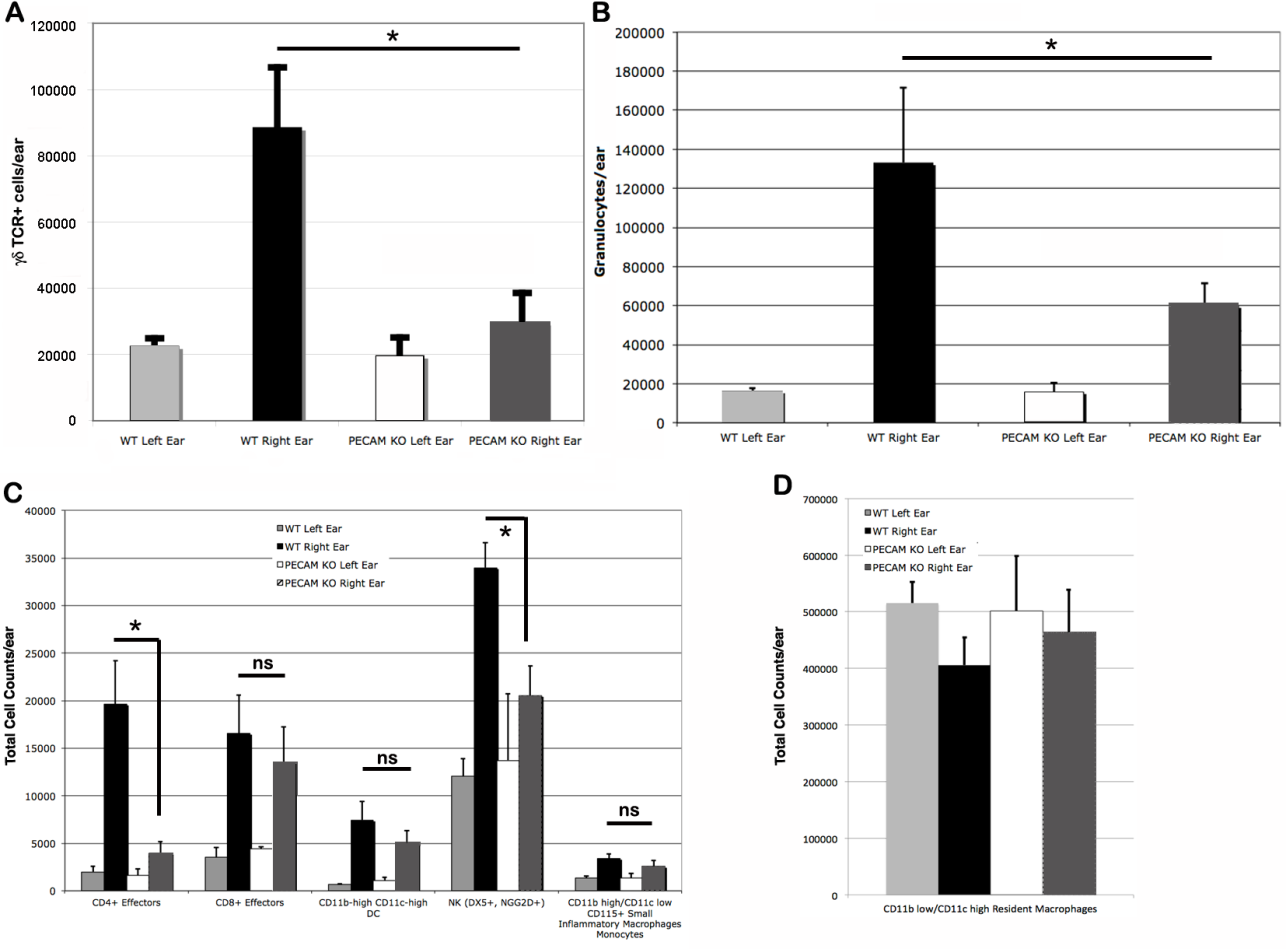
Cell counts from treated (right) and untreated (left) ears. Larger populations (A, B, and D) are separated out for clarity. (A) γδ TCR+ cell counts. (B) Granulocytes. (C) Other lymphocyte populations. (D) Resident macrophages, by far the largest population, were largely unaffected by DNFB treatment. ^∗^*p* < 0.05, Tukey Kramer HSD test. ns, not significant difference.

Upon the secondary challenge five days later in the ear, ear redness and swelling were not significantly different between wild type and PECAM-1 deficient mice (Fig. 4). However, the observed number of inflammatory cells was significantly lower in PECAM-1 deficient mice (Fig. 5). γδ TCR+ cells (Fig. 6A), neutrophils (Fig. 6B), dendritic cells, DX5+/NKG2D+ cells, and classical CD4+ and CD8+ cells (Fig. 6C) were much lower overall. “Resident” macrophages (Fig. 6D) appeared to be mostly unchanged and were by far the largest population. PECAM-1 deficiency significantly reduced the influx of some populations (effector CD4+ CD62L−/CD44+ T cells, NK cells, neutrophils, and γδ TCR+ cells) but not others (effector CD8+ CD62L−/CD44+ T cells, and dendritic cells). It was most striking that the cells most mobilized by the secondary inflammatory response (neutrophils and γ δ TCR+ cells) were also most affected by PECAM-1 deficiency.

## Discussion

This study shows two important results. First, the high participation of DX5+/NKG2D+ phenotype cells in the primary immune response to DNFB does not necessarily correspond to a similarly proportioned response at a secondary challenge. Likewise, γδ TCR+ cells were relatively minimal (∼1% of total) in the primary challenge site but became quite vigorously involved in the secondary response, outnumbering any other single population (other than resident macrophages) by almost 5-fold.

Second, PECAM-1 deficiency manifested itself in differential effects on each population. Most were affected by the deficiency, but it was surprising that CD8+ T effectors were able to overcome the lack of PECAM-1 whereas CD4+ T cell infiltration was clearly altered. We did not look for CD4+ CD3+ T cells at the primary exposure site, as we did not expect to find many in the skin as we thought the primary response would be largely driven at this stage by macrophages and NK cells, and that CD4 responses would be activated later by antigen-presenting cells. Further, it would be fascinating to examine the effects on CD4+ T cells at both the primary skin flank exposure site and the ear as CD4 cells do play a significant helper role in the response (Saint-Mezard et al., 2005). Clearly CD4 help at both sites may potentially drive some activation and differential effects on CD8 and γδ TCR+ cells at both sites. DX5+/NKG2D+ cells, being innate, may in turn be waning in their responses as the adaptive immune response takes over the inflammatory process.

Another leukocyte population we did not examine is the Innate Lymphoid cells (ILCs), which were largely unknown at the time these studies were performed (Walker, Barlow & McKenzie, 2013). NK cells are also part of the Innate Lymphoid cell family, and it makes logical sense to explore the role of ILC1, ILC2, and ILC3 subsets in this response. Additionally, we did not look at cell counts for fibroblasts, epithelial cells, endothelial cells, which may comprise a large number of the total cells collected (Fig. 3A). A more holistic approach to all of the cells involved could be very informative as it was also somewhat surprising to see the differential effects on the vasculature dilation (Fig. 1) in PECAM-1 deficient strain at the site of primary exposure. As PECAM-1 is vital to endothelial cell permeability (Graesser et al., 2002; Lishnevsky et al., 2014), this study shows quite dramatically the role of this molecule to the earliest stages of the inflammatory response in this model as well.

There is considerable evidence that γδ TCR+ cells play a significant role in skin allergies and hapten-induced contact sensitivity (Nielsen et al., 2014). Many of them also expressed NKG2D, and NKG2D played a role in the activation cycle (Nielsen et al., 2015). More recently, the contribution of Vγ4 T cells in the response to DNFB and subsequent neutrophil infiltration was elegantly mapped out by Jiang et al. (2017). Our work largely complements those studies, though it is important to note that their use of the term “primary exposure” still is measured after a second exposure to DNFB.

Our studies do pose many questions to answer in possible future studies. In particular, we would like to pursue a longer lag time between primary and secondary challenges. For consistency, we used the classical assay used by many other groups with a total of 7 days between primary challenge and harvest after secondary challenge (Asherson & Ptak, 1968; O’Leary et al., 2006; Warfvinge & Larsson, 1991). Perhaps the response kinetics would be altered between each population. It has previously been shown that memory recall response by NK cells in mice can last as long as four months (Paust et al., 2010).

We would also would have preferred to use NK1.1 as a marker for confirmation of the NK phenotype. Unfortunately, we did not use NK1.1 on FVB/n mice as we only retrospectively discovered that FVB/n mice do indeed express NK1.1 (Liu et al., 2000). Finally, it would be very important to identify which γδ TCR population(s) are mobilized, as there is tissue specific localization of the γδ TCR subsets. The Vγ3 subset is commonly found in skin (Jin et al., 2010), however it would be interesting to assess whether any blood Vγ1, Vγ2 or epidermal Vγ5 (Nakamura et al., 2013) would be mobilized or differentially affected by PECAM-1 deficiency, similar to the different effects on CD4+ and CD8+ T cells.

Unfortunately, this strain of mice was lost to future studies at this time due to poor breeding and limited lifespan due to spontaneous pulmonary fibrosis (Early et al., 2009). There is a PECAM-1 deficient mouse in the C57BL/6 background. The C57BL/6 surprisingly has a near-normal response in many inflammatory models compared to wild type mice (Duncan et al., 1999), whereas the FVB/n strain exhibited the phenotype we expected with markedly reduced inflammatory responses. This difference mapped to a locus on chromosome 2 (Seidman et al., 2009). The C57BL/6 strain is also resistant to the pulmonary fibrotic disease (Schenkel et al., 2006), and this appears to be related to vascular function (Lishnevsky et al., 2014). It would be interesting to again compare and contrast these strains and the effects on their populations in future studies. However, we believe any subsequent studies would be best conducted with cell-targeted gene disruption of various subsets like monocytes, natural killer lymphocytes, or γδ TCR+ to also further determine the roles of these subsets in the response to DNFB.

##  Supplemental Information

10.7717/peerj.3555/supp-1 Data S1 Ear Experiments 1–3 + Skin Experiment 1 Notebook and raw data Complete lab notebook notes and raw data from first 3 sets of experiments on ears (secondary exposure after primary sensitization) and first skin digest experiment after discovering the vascular changes in the PECAM-1 deficient mice.

10.7717/peerj.3555/supp-2 Data S2 Ear Experiment 4 + Skin Experiments 2–4 Notebook and raw data Fourth ear experiment and skin primary exposure notebook and raw data from experiments 2–4.

## Additional Information and Declarations

### Data Availability

The following information was supplied regarding data availability: The raw data has been supplied as Datas S1 and S2.
