# Supplementary material for: Differential effect of Platelet Endothelial Cell Adhesion Molecule-1 (PECAM-1) on leukocyte infiltration during contact hypersensitivity responses
Source: PeerJ. 2017 Jul 10;5:e3555. doi: 10.7717/peerj.3555 (PMC5507171; doi:10.7717/peerj.3555)
Supplement: Data S1 — Complete lab notebook notes and raw data from first 3 sets of experiments on ears (secondary exposure after primary sensitization) and first skin digest experiment after discovering the vascular changes in the PECAM-1 deficient mice. [file peerj-05-3555-s001.pdf]

5/4/06

			Right (Rx)	Left (CTRL)	Δ
WT	39056	♂	1.2	1.0	0.2
		♀	0.95	0.65	0.3
KO	47007	mark	1.3	0.8	0.5
		unm	1.4	1.2	0.2
KO	48058		1.8	1.5	0.3

DNF8 I

5/5/6

			R	L	Δ
WT	39056	♂	1.4	1.2	0.2
		♀	1.8	1.0	0.8
KO	47007	marked	1.7	1.1	0.6
		unmarked	1.6	1.1	0.5
KO	48058		1.7	1.3	0.4

National® Brand 42-381 50 SHEETS EYE-EASE® 5 SQUARE 42-382 100 SHEETS EYE-EASE® 5 SQUARE 42-389 200 SHEETS EYE-EASE® 5 SQUARE

		tissue	vessels
47007 U	R ear		
	L ear		
47007 M	R ear		
	L ear		
48088	R ear		
	L ear		
39056	R ear		

National® Brand
42-381 50 SHEETS EYE-EASE® 5 SQUARE
42-382 100 SHEETS EYE-EASE® 5 SQUARE
42-389 200 SHEETS EYE-EASE® 5 SQUARE

5/26/06

6 CD44 + α rat [illegible] + SA 488 + CD8 [illegible] 647
R rat
+ ~~mouse~~ serum
block

1 neg ctrl

CD49b + α rat [illegible] block + SA PE Cy5.5 + CD11b FITC

National® Brand 42-381 50 SHEETS EYE-EASE® 5 SQUARE 42-382 100 SHEETS EYE-EASE® 5 SQUARE 42-389 200 SHEETS EYE-EASE® 5 SQUARE

Ear Nuclei Staining — dermis & collagen

47007 U R ear ~~20830~~ ~~12270~~ L ear

8x9 27292 72 in^2 | 4x18 ~~9271~~ 22682

5.5x5.5 31925 30.25 in^2 | 5.5x5.5 10326

39056 M R ear ~~24258~~ ~~6023~~

48x18 31064 72 in^2 | 4x9 9849 33 nuclei

5.5x5.5 13459 30.25 in^2 | 5.5x5.5 11911 Conv[illegible]

47007 M R ear ~~22235~~ 30263

8x9 52945 72 in^2 | ~~4x9 7849 33 nuclei~~

5.5x5.5 25703 30.25 | ~~4874 [illegible]~~

2.5x9 8837

— how many pixels/nuclei

48058 — need untreated numbers

4x18 47308 72 in^2 | 21+15

5.5x5.5 8358 30.25 in^2 | ~~[illegible]~~

~~[illegible]~~ 40 nuclei

22416

39056 F R ear

4x18 20153 72 in^2 | 8x9 15874

~~[illegible]~~ 7.5x4 8165 30.25^2 | 7.5x4 6252

~300 pixels/nucleus

National Brand 42-381 50 SHEETS EYE-EASE 5 SQUARE 42-382 100 SHEETS EYE-EASE 5 SQUARE 42-389 200 SHEETS EYE-EASE 5 SQUARE

Oneway Analysis of right Ear d1 By Column 6

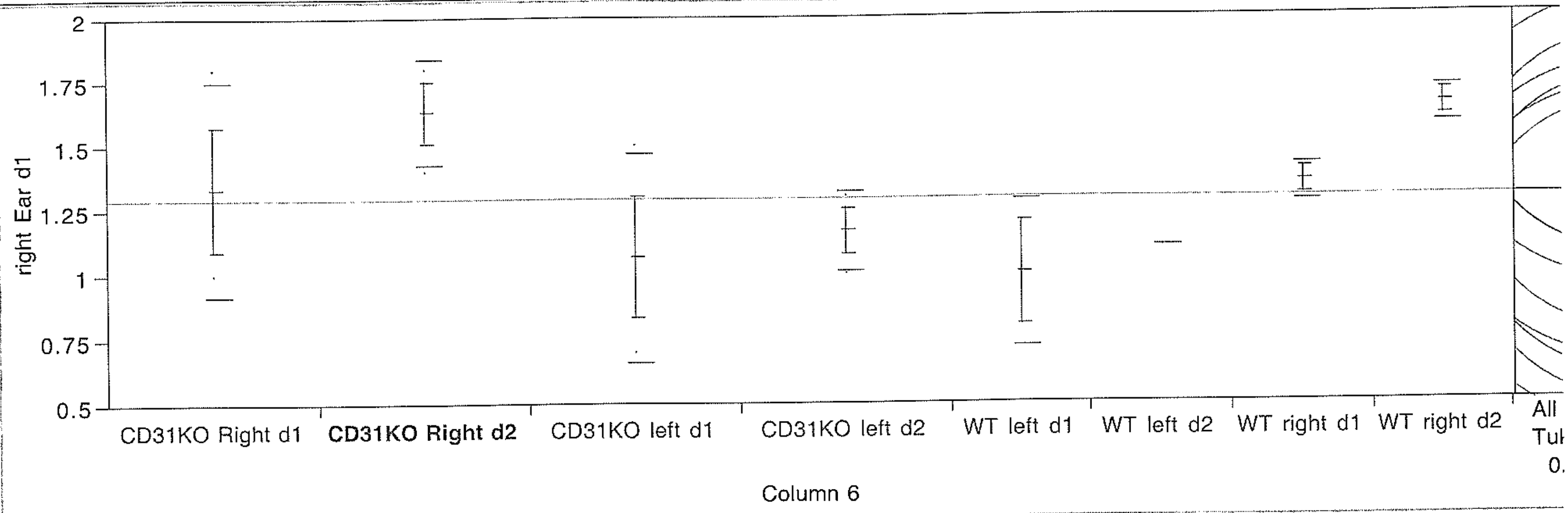

Means and Std Deviations

Level	Number	Mean	Std Dev	Std Err Mean	Lower 95%	Upper 95%
CD31KO Right d1	3	1.33333	0.416333	0.24037	0.299	2.3676
CD31KO Right d2	3	1.63333	0.208167	0.12019	1.116	2.1504
CD31KO left d1	3	1.06667	0.404145	0.23333	0.063	2.0706
CD31KO left d2	3	1.16667	0.152753	0.08819	0.787	1.5461
WT left d1	2	1.00000	0.282843	0.20000	-1.541	3.5412
WT left d2	2	1.10000	0.000000	0.00000	1.100	1.1000
WT right d1	2	1.35000	0.070711	0.05000	0.715	1.9853
WT right d2	2	1.65000	0.070711	0.05000	1.015	2.2853

almost identical

Oneway Analysis of Total cell counts By Group

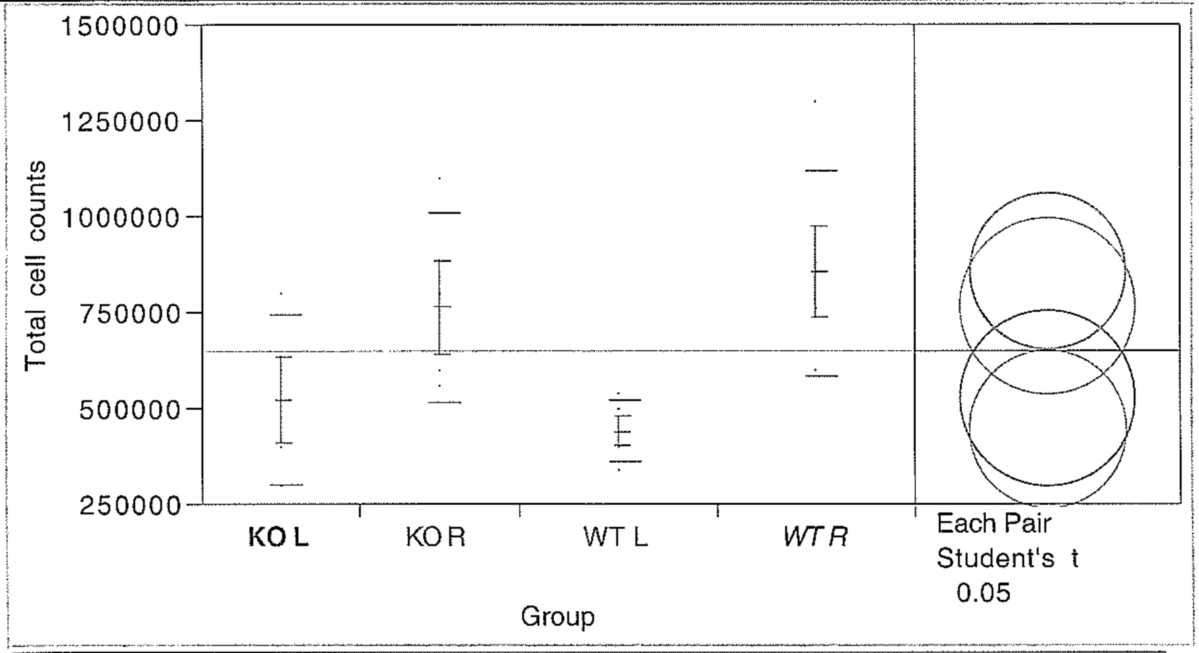

Means and Std Deviations

Level	Number	Mean	Std Dev	Std Err Mean	Lower 95%	Upper 95%
KO L	4	525000	221736	110868	172169	877830.79
KO R	4	765000	246779	123390	372319	1157680.9
WT L	5	444000	79246	35440	345603	542397.47
WT R	5	856000	267357	119566	524032	1187968.1

looks nearly identical
to manual scoring on [illegible]

From 7/7/06

7/7/06 | Ear thickness

PNF3 II

24 hrs (mon) | 48 hrs ([illegible])

~~51774~~ 5

			24 hrs L	24 hrs R	48 hrs L	48 hrs R	
Ko1	• 51574		~~L = .9~~	~~R = 1.1~~			
			L = 1.1	R = 1.55	L = 0.9	R = 1.6	Lungs Marginal
WT1	• 52455	#4	L = 1.2	R = 1.35	L = 0.9	R = 1.2	
Ko2		#3	L = 1.6	R = 2.2	L = 1.4	R = 1.3	
WT2	51587	#1	L = 1.25	R = 1.6	L = 1.0	R = 1.5	
WT3		#2	L = 1.3	R = 2.2	L = 1.3	R = 1.4	
					L = 1.0	R = 1.5	
Ko3	52453	#2	L = 1.3	R = 2		R = 1.4	Lungs = "Look Like Shit"
WT4		#3	L = 0.9	R = 1.65	L = 1.4	R = 1.5	
Ko4	51254	#3	L = 1.9	R = 0.9	L = 1.1	R = 1.5	
WT5		#4	L = 0.7	R = 1.6	L = 1.35	R = 1.2	

42-381 50 SHEETS EYE-EASE® 5 SQUARE
42-382 100 SHEETS EYE-EASE® 5 SQUARE
42-389 200 SHEETS EYE-EASE® 5 SQUARE
National® Brand

7/7/06

~~51574~~		5		
51574		~~L = .9~~	~~R = 1.1~~	
		L = 1.1	R = 1.55	K01
52455	#4	L = 1,2	R = 1.35	WT1
	#3	L = 1.6	R = 2.2	K02
51587	#1	L = 1,25	R = 1,6	WT2
	#2	L = 1,3	R = 2.2	WT3
52453	#1	L = 1,3	R = 2	K03
	#3	L = 0.9	R = 1,65	WT4
51254	#3	L = 1,9	R = 0.9	K04
	#4	L = 0.7	R = 1,6	WT5

National Brand

1μg for 1e6 cells

7/7/06 0.1μg for 1e5 cells

CD4 PE-Cy5 or CD8 PE-Cy5 0.2mg/ml 10μl
CD44 PE 0.2mg/ml 10μl
CD62 APC 0.2mg/ml 10μl 120

CD11a PE-Cy5 0.1mg/ml so 1μl for 1e5 cells × 20 = 20μl
IgD PE 0.2mg/ml 0.5μl 10μl
CD62 APC 0.2mg/ml 10μl 155
CD8 FITC 0.5mg/ml 4μl

CD11b FITC 0.5mg/ml → PE Cy7 4μl
CD11c [illegible] 0.2mg/ml biotin PE Cy5 10μl 128
GR1 APC 0.2mg/ml 10μl
CD115 PE

CD45R PE 0.2mg/ml 10μl
CD8 PE-Cy5 0.2mg/ml 10μl 175
F4/80 FITC 0.5mg/ml 4μl

PE Cy7
CD11c - PE Cy5
F4/80 FITC
GR1 - APC
CD115 PE

Next time

7/7/06

4
8
B
DC
NK
(CO5FKC?)

ALL FUBIN
ONFB CHS @ 48hr

~~[illegible]~~

KO	KO	KO	KO	KO
1L	1L	1L	1L	1L
4	8	B	DC	NK

~~KO1R4~~

KO1R KO1L
KO2R KO2L
~~KO~~ KO3R KO3L
KO4R KO4R
WT1R WT1L
WT2R WT2L
WT3R WT3L
WT4R WT4L

7/7/06 liver samples

→ more T cells here

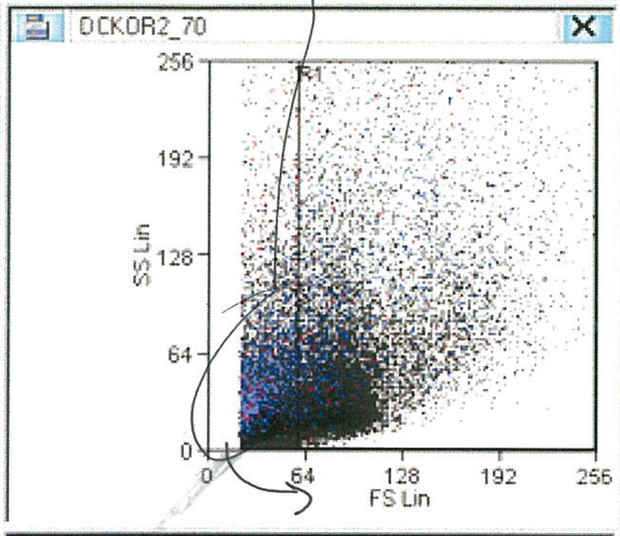

Re...	Count	% Hist	% All	Mean
Total	49093	100.00	100.00	67.13, 46.13
R1	25050	51.03	51.03	96.30, 62.09

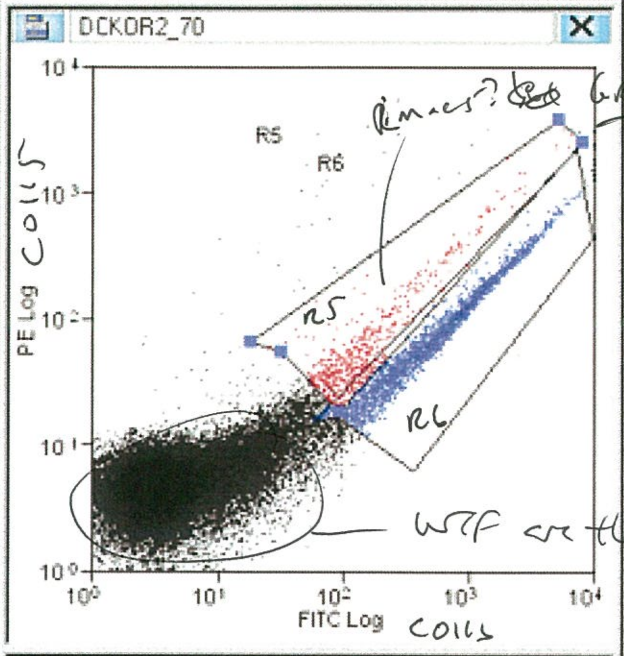

Re...	Count	% Hist	% All	Mean
Total	49093	100.00	100.00	67.46, 15.48
R5	615	1.25	1.25	287.90, 123.11
R6	3214	6.55	6.55	832.16, 108.39

Skin fibroblasts? <10% lymphocytes

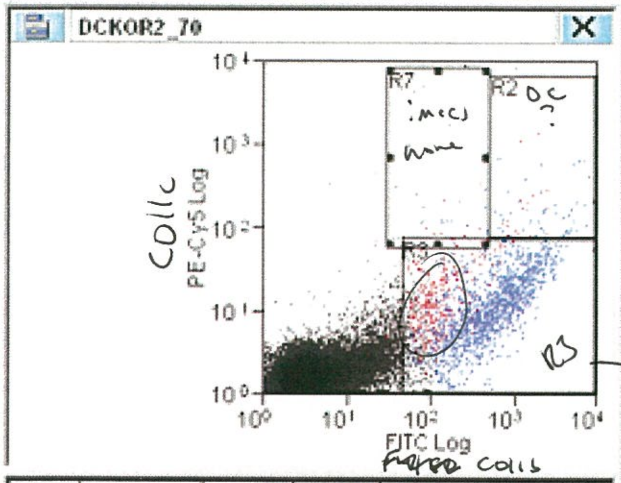

Re...	Count	% Hist	% All	Mean
Total	49093	100.00	100.00	67.46, 6.02
R7	93	0.19	0.19	190.45, 430.88
R2	207	0.42	0.42	3145.34, 272.29
R3	4474	9.11	9.11	555.37, 13.85

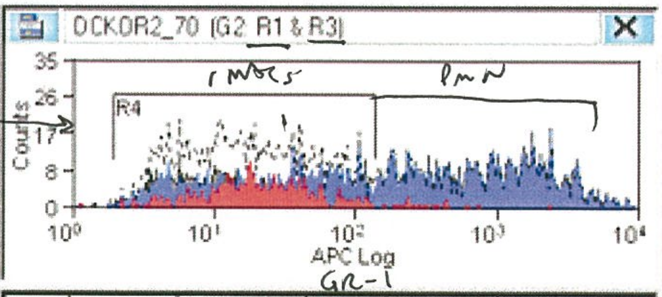

R...	Count	% Hist	% All	Mean	Median
Total	2272	100.00	4.63	552.92	52.33
R4	1384	60.92	2.82	29.21	16.55

— no compensation

— look at F4/80 vs DX5/CD49b

— since so many GR1+/CD11b do diff for eosinophils!

\BKOL1_47.fcs

L1_47

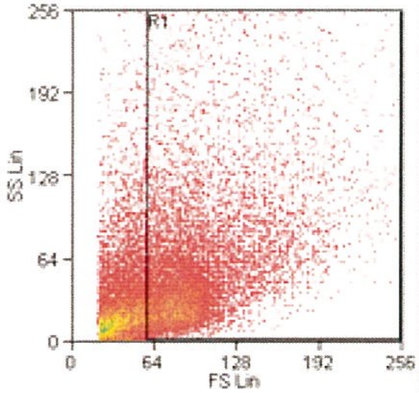

Re...	Count	% Hist	% All	Mean
Total	33235	100.00	100.00	67.85, 41.72
R1	17473	52.57	52.57	95.16, 56.12

BKOL1_47 (G4: R7)

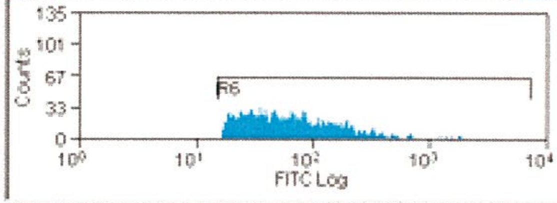

R...	Count	% Hist	% All	Mean	Median
Total	1592	100.00	4.79	121.06	58.29
R6	1592	100.00	4.79	121.06	58.29

BKOL1_47 (G5: R1 & R8)

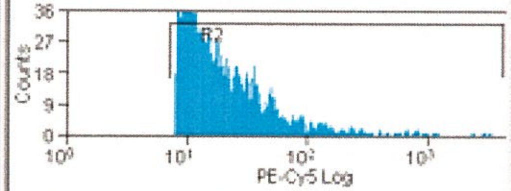

R...	Count	% Hist	% All	Mean	Median
Total	1301	100.00	3.91	48.76	15.96
R2	1301	100.00	3.91	48.76	15.96

BKOL1_47

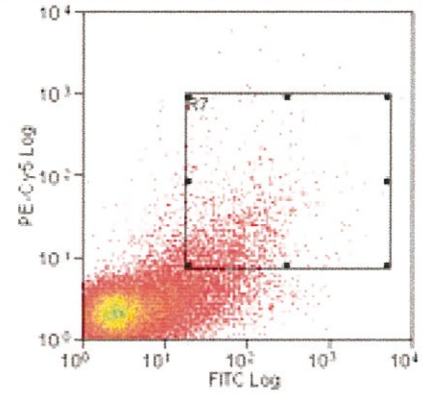

Re...	Count	% Hist	% All	Mean
Total	33235	100.00	100.00	13.52, 5.55
R7	1592	4.79	4.79	121.06, 36.21

BKOL1_47 (G4: R7)

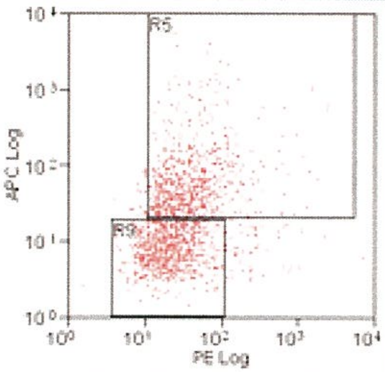

Re...	Count	% Hist	% All	Mean
Total	1592	100.00	4.79	61.43, 76.17
R5	733	46.04	2.21	74.08, 153.96
R9	817	51.32	2.46	25.18, 8.94

BKOL1_47 (G5: R1 & R8)

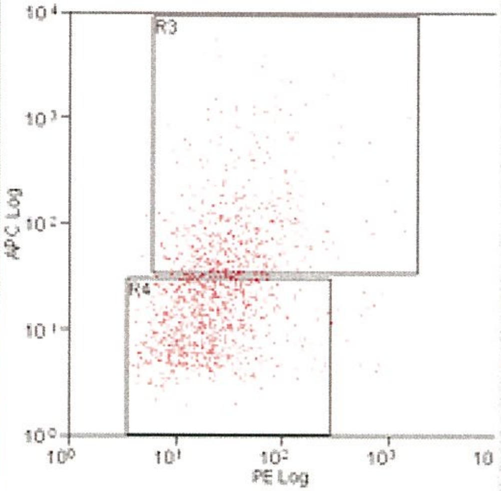

Re...	Count	% Hist	% All	Mean
Total	1301	100.00	3.91	47.35, 95.89
R3	495	38.05	1.49	66.76, 210.90
R4	782	60.11	2.35	28.29, 12.88

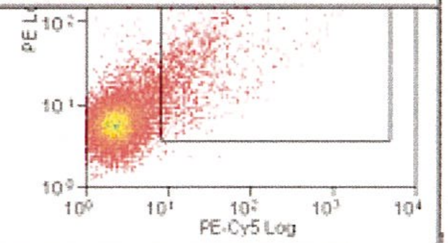

Re...	Count	% Hist	% All	Mean
Total	17473	100.00	52.57	6.64, 10.22
R8	1301	7.45	3.91	48.76, 47.35

BKOL1_47 (G4: R7)

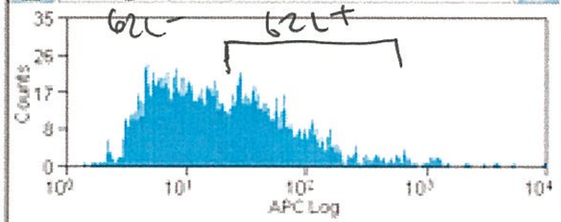

R...	Count	% Hist	% All	Mean	Median
Total	1592	100.00	4.79	76.17	17.15

3/31/06 [illegible]

7/31/06

48 × 50 = 00

28 × 50 = 1400

30 × 50 = 1500; 1500 − 30 = 1470

1400 ul total
− 28
1372

30

K1L	40 × 10^4 × 2	= 8 × 10^5 total	735	12.5 ml
K2L	15 × 10^4 × 4	= 6 × 10^5 total		100 ml
K3L	15 × 10^4 × 2	3 × 10^5 total		300 ml — short 80 1/5
K4L	10 × 10^4 × 4	= 4 × 10^5 total		200 ml
W1L	20 × 10^4 × 2	= 4 × 10^5 total		200 ml
W2L	17 × 10^4 × 2	= 3.5 × 10^5		300 ml
W3L	22 × 10^4 × 2	= 4.5 × 10^5		200 ml
W4L	25 × 10^4 × 2	= 5 × 10^5		200 ml
W5L	27 × 10	= 5.5 × 10^5		200 ml
W5R	38 × 10 × 2	= 7.6 × 10^5		150 ml
W1R	30	6 × 10^5		100 ml
W2R	37	7.4 × 10^5		150 ml
W3R	44	9 × 10^5		100 ml
W4R	1.5	1.3 × 10^6		100 ml

K1R	53	1.1 × 10^6	100 ml
K2R	28	5.6 × 10^5	200 ml
K3R	41	8 × 10^5	125 ml
K4R	31	6 × 10^5	100 ml

% of total →	R1 (!maces) MΦ	R2 mDC / PMN	R3 [illegible] / ~~DC~~	% GR1 in R3 R4	~~DC~~ ~~R2~~	NK Ky160+	Y<b+/8-	Y5b+/8+
WL1	0.3	0.15	~~0.32~~ 4.5 4.5	~~4.5~~ 90 90	~~0.45~~	5	3.7	0.5
WL2	0.7	0.13	4	87		3.9	2.6	0.2
WL3	0.3	0.12	2.9	~~86~~ 93		3.1	1.9	0.16
WL4	0.2	0.2	3.4	89		2.6	1.5	0.13
WL5	0.4	0.3	3.7	96		3.2	2.2	0.25
WR1	0.7	0.8	6.2	91		5.7	5.5	0.2
WR2	0.3	0.8	18.6	53		9.9	5.6	0.6
WR3	0.5	0.8	12.4	60		6.4	4.0	0.3
WR4	0.3	1	21	52		14.4	2.7	0.6
WR5	0.3	0.5	14.2	57		~~14~~ 7.6	3.3	0.4
KL1	0.3	0.17	3.3	89		4.8	4.2	0.2
KL2	0.3	0.3	3.5	85		4.2	2.2	0.13
KL3	0.1	0.1	2.3	87		2.5	1.1	0.3
KL4	0.2	0.2	2.3	72		2.7	1.2	0.5
KR1	0.3	0.5	8.0	62		6.2	2.7	0.8
KR2	0.2	0.4	9.1	62		6.4	3.4	0.3
KR3	0.5	1.1	6.8	71		6.8	2.	0.3
KR4	0.3	0.5	6.7	68		6.2	2.5	0.3

7/11/06 analysis of 7/7/06 Exp stain

	4				8				13 1 (cos+)			13 coll' 82		
		62+	62/44	44+					60+	cos2+	62-		62+	62-
WT 1L	1%	.46 .41	1 .08	40 .35	1.5	48	5	44	~~4.8~~	~~46~~	~~51~~	~~3.9~~	~~38~~	~~60~~
2L	0.8	60	3	30	1.1	41	9	38	~~2.4~~	~~36~~	~~60~~	~~2~~	~~28~~	~~30~~
3L	.35	45	9.5	40	1.4	44	4	46	~~2.2~~	~~32~~	~~41~~	~~1.8~~	~~32~~	~~66~~
4L	1.5	31	16	55	2.3	25	6	65	6.4	15	74	0.3	13	86
5L	.84	44	10	38	1.8	45	7	34	3.7	32	61	2.8	28	71
1R	2.8	28	14	65	1.8	21	3	72	3.9	34	65	3	21	77
2R	4	40	18	48	3.4	26	7	69	1.6	26	72	1	17	80
3R	~~3.7~~	40	16	54	2.3	27	6	59	6.4	22	70	4.4	21	76
4R	5.7	40	10	50	5.6	48	8	43	3.4	27	74	4.7	22	72
5R	4.1	32	6.5	54	3.1	26	4	61	6.7	28	72	3.5	24	74
Ko 1L	0.7	55	3	38	2	59	10	37	4.8	46	51	3.5	38	60
2L	1.3	27	18	51	2.4	21	8	66	2.4	36	60	2	28	30
3L	0.5	23	22	55	0.9	21	6	69	2.2	42	49	1.8	32	65
4L	0.7	51	21	32	1.2	37	6	42	3	34	60	2.4	25	71
1R	1.1	42	18	35	2.3	24	4	60	6	25	69	4	23	74
2R	0.6	43	12	36	1.8	27	6	51	3	29	69	2	28	76
3R	1.7	28	20	51	4.1	24	4	70	0.9	15	82	0.5	9	89
4R	1.6	47	17	36	3.2	27	6	56	9	36	65	3.6	24	74
WT 1L									3.4	45	52	2.8	33	65
2L									3.2	29	68	2.3	21	77
3L									2.9	40	37	2.4	30	69

7/11/06 Analysis of 7/7/06 expt

little
- up class switching
- evidence for CoGZ as activator?

3/13/07 DNFB ear III

DNFB III

		R	L
WT	58801	1.8	0.7
		1.8	1.3
		1.6	0.7
		2.1	1.2
WT	83979	1.6	1.2
		2.4	1.3
KO	88034	2.2	1.2
KO	58804	2.4	1.3
		1.7	1.2
		1.6	1.2
		1.9	1.6

ear thickness

National® Brand 42-381 50 SHEETS EYE-EASE® 5 SQUARE 42-382 100 SHEETS EYE-EASE® 5 SQUARE 42-389 200 SHEETS EYE-EASE® 5 SQUARE

3/14/07 Analysis lung

CD4		naive	$44^+/62L^+$	effector
FW1	31%	55	21	24
~~[illegible]~~ lymphoma? FW2	8	54	25	22
FW3	28	57	20	22
FW4	34	62	19	19
lymphoma? FW5	4	58	22	19
FW6	23	54	22	25
FK1	26	54	40	8
FK2	21	69	~~39~~ 29	4
FK3	11	26	55	19
FK4	16	67	30	5
FK5	12	70	23	5

CD8					γδ
FW1	8	35	60	11	32
FW2	3.5	46	51	8	30
FW3	5	43	48	13	26
FW4	7	43	52	11	26
FW5	8	32	61	15	30
FW6	8	27	65	17.5	38
FK1	5	39	57	9	20
FK2	4	37	56	5	22
lymphoma FK3	0.5	21	59	17	5
FK4	4	51	39	6	19
lymphoma FK5	0.4	44	47	6	4.3

DC/mφ	images R1 (CD11b+, CD11c+, GR1hi)	R2 OC	R3	PMN GR1low	PMN GR1high
FW1	8	2	16	31	67
FW2	8	2	13	27	69
FW3	8	2	10	25	71
?→ FW4	0.7	0.2	1.5	23	74
?→ FW5	10.5	3	13	30	67
FW6	7	2	11	33	62
FK1	1	0.13	3	34	59
FK2	10	2	13	29	68
FK3	13	3	5	48	44
FK4	18	3	8	28	68
FK5	6	2	14	19	78

NK	DX5+, CD3	DX5+, CD3+	of all ungated CD3+	F4/80+
FW1	12	2	14	29
FW2	16	2	19	29
FW3	22	25	18	29
FW4	27	3	18	32
FW5	20	4	18	38
FW6	26	8	10	38
FK1	5	23	26	35
FK2	2	0.7	5	16
FK3	7	2	8	45
FK4	9	2	15	49
FK5	7	2	8	20

4	3
~~15~~	20
~~21~~	13
~~14~~	14
~~20~~	15
70	62
17.5	15.5

Ecr counts

56	40
43	40
50	38
~~5~~ 5×10^5	4×10^5

16.5
.2
3.30

6.6
.2
1.32

WRE 1 3.0×10^5/ml
2 3.0×10^5/mL
3 4×10^5/mL
4 2.2×10^5/mL
5 3.5×10^5/mL
6 1×10^5/mL

KRE 1 1×10^5/mL
2 2×10^5/mL
3 8×10^4/mL
4 1×10^5/mL
5 1.2×10^5/mL

National® Brand 42-381 50 SHEETS EYE-EASE® 5 SQUARE
42-382 100 SHEETS EYE-EASE® 5 SQUARE
42-389 200 SHEETS EYE-EASE® 5 SQUARE

3/14/07

Skin counts			Lung	
FK1	18 x10 x10[illegible]	1.8x10[illegible]	10	1x10[illegible]
FK2 ~~FK2~~	40 ~~8~~ x 10 x10[illegible]		16	1.6x10[illegible]
FK3	6		12	1.2x10[illegible]
FK4	2		30	3x10[illegible]
FK5	4		6	6x10[illegible]
FW1	4		1e6	
FW2	2		8e5	
FW3	8		5e5	
FW4	<1 3x10[illegible]		1e6	
FW5	<1		4 x105	
FW6	5		2e5	

Cell Survey 3/14/07

Previously on 3/8/07 Applied 10ul of DNFB to flanks of 6 WT mice + 5 KO (PECAM)

On 3/12/07 applied 10ul to (R) ear of all 11 mice and 10ul of acetone + olive oil to (L) ears.

3/14/07

National® Brand 42-381 50 SHEETS EYE-EASE® 5 SQUARE 42-382 100 SHEETS EYE-EASE® 5 SQUARE 42-389 200 SHEETS EYE-EASE® 5 SQUARE

42-381 50 SHEETS EYE EASE 5 SQUARE
42-382 100 SHEETS EYE EASE 5 SQUARE
42-389 200 SHEETS EYE EASE 5 SQUARE
National Brand

Color

PE Cy5	CD4	CD8	CD19	CD11c	
PE	CD44	CD44	IgD	CD115 (M-CSF)	CD 495
APC	CD62L	CD62L	CD62L	Gr1	CD3 (APC Cy7)
FITC				CD115	F4/80

1. 50ul blood per tube in 1 ml PBS (no cations). Split into two tubes, label clearly. Add 2ul rat serum/tube, can be in PBS.
2. Add 200ng of each antibody:

B Test tube
D011 FITC 2ul/tube
H2Dd PE 1ul
CD31 biotin (will add Steptavidin [SA] APC later) 0.400ul

26 D011 52ul
26ul
10.4ul

16 [illegible] KO 6.4

3. Incubate cold and dark 30 minutes minimum
4. Wash in PBS (no cations) 3 X
5. Add SA APC (1 ul/tube)
6. Incubate cold and dark 30 minutes minimum
7. Wash 1X in PBS
8. Lyse in 1800ul distilled water until clear, add 200ul 10X PBS
9. Wash in PBS (no cations) 2 X
10. Run on flow cytometer

DO11 = 1ul/tube 100ug/mL .1mg/mL

.5ul/tube

DD4 PECy5 .5/tube x 33 = 16.5 >100

3 x CD62L APC .5/tube x 33 = 16.5 49.5 tot - >200

2 x CD44 PE .5/tube x 33 = 16.5x2 = 33 tot 33 16.5 [illegible] >100

CD8a PECy5 a? .5/tube x 33 - 16.5

~~CD62L~~ .2/tube = 6.6

Pangamma δ FITC ~~1/tube x 33 = 33 ul~~

CD19 PECy5 1/tube x [illegible] 33 [illegible]0

~~[illegible]~~

IgD .5/tube x [illegible] 16.5 [illegible]200

CD11b FITC .2/tube x 33 = 6.6 ~~[illegible]~~ 15

CD11c PECy5 .5ul/tube x 33 = 16.5ul barely enough

CD115 PE .5/tube x 33 = 16.5 <14 NOT ENOUGH

Gr-1 APC .[illegible] x 33 = 16.5 >100

CD49b Pan NK

F4/80 FITC .2ul/tube x 33 = 6.6 >200

CD3e? PC-Cy-7 .5/tube x 33 = 16.5 >100

~~CD44d? .2/tube x 33 = 6.6~~ = 70 ul 16.5 x 3 = 49.5

x 11

					Have		
.7	CD4 PE-Cy5	.5/tube x 33	= 16.5	15	>1000	3.5	2.5 2.5
"	CD62L APC	.5/tube x 33	= 16.5	15	>200	3.5	2.5
"	CD44 PE	.5/tube x 33	= 16.5	15	>100	3.5	
.7	CD8 PE-Cy5 w?	.5/tube x 33	= 16.5		69	3.5	2.5
"	CD62L APC		16.5		>200	3.5	2.5
"	CD44 PE		16.5		>1000	3.5	2.5
	Pan gamma delta FITC	in foil?	need 6.6			1.5	1.0
	CD19 PE-Cy5	1/tube x33	= 33		~70		
	CD62L APC		16.5		>200		
	IgD PE	.5/tube x 33	= 16.5		>200		
	CD11b FITC	.2/tube x 33	= 6.6		~15 barely enough.	1.5	1.0
	CD11c PECy5	.5 tube x 33	= 16.6			3.5	2.5
	CD115 PE	.5/tube x 33	= 16.5		<14 lose all	3.5	2.5
	Gr-1 APC	.5/tube x 33	= 16.5		>100	3.5	2.5
	CD49b PE	.5/tube x 33	= 16.5			3.5	2.5
	F4/80 FITC	.2/tube x 33	= 6.6		>200	1.5	1.0
	CD3 APC-Cy7 NK	.5/tube x33	= 16.5		>100	3.5	2.5

.1 x 22 = 2.5
2.5

make each

0.7 x 22 = ~~1.54~~ 15.4 15
15.4 15

22
.2
4.4 5

1 ~~2200~~
22
50
1100

1,1ml + 100µl

CD4 PE-Cy5 6 } 1.25ml
CD62L APC 6
CD44 PE 6

CD8 PE-Cy5 6
CD62L APC 6
CD44 PE 6
pan γδ FITC 3

CD11b FITC 3
CD11c PeCy5 6
CD115 6
Gr1 6

CD49b 6
F4/80 3
CD3 6

National® Brand 42-381 50 SHEETS EYE-EASE® 5 SQUARE 42-382 100 SHEETS EYE-EASE® 5 SQUARE 42-389 200 SHEETS EYE-EASE® 5 SQUARE

DNFB ears 3/15/07

CD4	% CD4	R3 % naive	R4 % "memory"	R5 % effector
FW1	1.6	76	1	21
FW2	2.12	64	.6	35
FW3	1.03	56	2	42
FW4	1.12	90	3	7
FW5	.9	88	9	4
FW6	3.4	50	10	41
FK1	1.7	65	5.5	30
FK2	1.2	57	3	40
FK3	1.14	39	12	49
FK4	.56	36	3	59
FK5	2.0	16	11	72

CD8	% CD8	CD8+ % 88	CD8- 88	R3	R4	R5
FW1	6.7	47	3	89	8	0
FW2	1.4	44	6	85	12	.2
FW3	.8	31	2	84	12	.1
FW4	.3	25	4	89	9	0
FW5	.24	16	2.5	80	17	.16
FW6	.15	36	10	93	5	0
FK1	.29	28	8	92	6.3	0
FK2	.39	32	5.6	89	9	0
FK3	.14	20	4	91	6	0
FK4	.14	17	3	79	7	0
FK5	.1	20	6	89	6	0

National® Brand 42-381 50 SHEETS EYE-EASE® 5 SQUARE 42-382 100 SHEETS EYE-EASE® 5 SQUARE 42-389 200 SHEETS EYE-EASE® 5 SQUARE

DNFB ear 3/15/07 DC's + NKs

rMN — rMACs

DC

	R2 iMACs	R3 DC	R4	R5 Gr1^hi	R6 Gr1^lo const
FW1	.02	.05	22	85	15
FW2	.05	.02	38	72	26
FW3	.01	.02	13	87	13
FW4	.02	.06	27	83	17
FW5	.10	.4	10	80	19
FW6	.09	.09	31	89	18
FK1	.03	.02	16	73	26
FK2	.04	.01	17	72	27
FK3	.01	.01	11	77	22
FK4	.03	.02	6	82	17
FK5	.02	.02	11	81	18

NK

	R11 F4/80+	R8 DX5+ NK	R9 DX5+ CD3+	R10 CD3+ T cells
FW1	23	.35	.03	.2
FW2	48	1.5	.2	.3
FW3	18	.33	.03	.2
FW4	30	1.9	.13	.2
FW5	15	.3	.02	.2
FW6	40	1.2	.13	.3
FK1	30	.81	.13	.3
FK2	22	.26	.03	.24
FK3	16	.2	.04	.3
FK4	12	.3	.03	.2
FK5	16	.3	.05	.24

Control Ears 3/15/07

CD4	R12 %CD4	R13 %naive	R15 %effector	R14 %memory
FW1	2.24	31	36	23
FW2	2.4	46	37	11
FW3	4.3	11	72	13
FW4	2.8	39	43	13
FW5	3.3	26	58	11
FW6	4.0	37	48	7
FK1	3.0	24	51	20
FK2	4.0	13	68	16
FK3	2.21	25	61	11
FK4	2.9	30	50	7
FK5	2.0	27	52	13

CD8	R16 %CD8	CD8+ %88 / CD8- 88	R13 naive	R15 effector	R14 memory
FW1	.74	19	13	69	0
FW2	1.0	20	9	66	4
FW3	3.4	10	5	76	11
FW4	1.4	17	7	79	3
FW5	4.4	21	10	73	8
FW6	2.3	15	8	77	9
FK1	8.0	24	26	40	30
FK2	6.0	10	10	72	6
FK3	2.2	7.4	23	63	7
FK4	2.0	8.6	11	56	3
FK5	2.2	3	14	57	10

DC	R2	R3	R4	R5	R6
FW1	.04	.33	1.0	~~13.42~~ 10	90
FW2	.03	.35	1.0	16	85
FW3	.02	.13	2.47	72	27
FW4	.01	.23	.75	59	40
FW5	.07	.33	1.9	73	26
FW6	.04	.56	1.23	30	70
FK1	.01	.61	3.4	60	38
FK2	.05	1.3	8.4	82	9
FK3	.09	.58	1.8	80	20
FK4	.04	.4	1.4	43	56
FK5	.02	.40	2.3	83	17

← look at!

Control Ear 3/15/07

NK	F4/50+ R11	R8	R9	R10
F W1	21	0	0	.16
F W2	35	.1	.06	.4
F W3	12	.02	.02	.17
F W4	32	.73	.24	.42
F W5	26	.23	.07	.51
F W6	21	.21	.01	.44
F K1	36	5.2	.6	.7
F K2	21	.20	.02	.24
F K3	17	.11	.02	.36
F K4	21	.06	0	.27
F K5	8	.08	.02	.23

42-381 50 SHEETS EYE-EASE® 5 SQUARE
42-382 100 SHEETS EYE-EASE® 5 SQUARE
42-389 200 SHEETS EYE-EASE® 5 SQUARE
National® Brand
