# Supplementary material for: Differential effect of Platelet Endothelial Cell Adhesion Molecule-1 (PECAM-1) on leukocyte infiltration during contact hypersensitivity responses
Source: PeerJ. 2017 Jul 10;5:e3555. doi: 10.7717/peerj.3555 (PMC5507171; doi:10.7717/peerj.3555)
Supplement: Data S2 — Fourth ear experiment and skin primary exposure notebook and raw data from experiments 2–4. [file peerj-05-3555-s002.pdf]

A B C E D/F

| CD4+ T | CD8+ T | CD19+ B | NK/NK T | Monocyte/DC | Color |
|---|---|---|---|---|---|
| CD3 | CD3 | CD3 (-) | CD3 | | APC CY7 |
| CD4 | CD8 | CD19 | CD8 | CD11c -out! | PE CY5 |
| CD44 | CD44 | CD5 | γδ T cell | CD115 – PE | FITC |
| CD62L | CD62L | CD62L | | CD11b FITC | APC |
| | | | | GR1 APC | PE Cy7 |
| FoxP3 | | | DX5 (CD49b) | | PE |
| | | IgD PE | | CD206-biotin | ~~PE~~ FITC |
| | | | CD1d | PE-Cy7 | APC |
| | | | | | PE Cy7 |

*Intracellular staining for ~~BrdU~~ and FoxP3*

Cells were surface stained as described, washed, and then fixedfor 5 min at 37° C in 4% paraformaldehyde. Cell suspensions were then permeabilized by incubating in PBS containing 0.1%Triton X-100 for 1 h on ice. For detection of BrdU incorporation,permeabilized cells were incubated for 10 min at 37°C in RPMI 1640 containing recombinant DNase. Afterward, suspensions were washed in incubated on ice for 40 min with anti-BrdU Absfollowing the manufacturer's protocol. Suspensions werewashed and then incubated overnight with anti-FoxP3 Abs in PBS containing 0.5% BSA and analyzed the following day by flow cytometry.For FoxP3 staining only, the BrdU relevant steps were omitted.

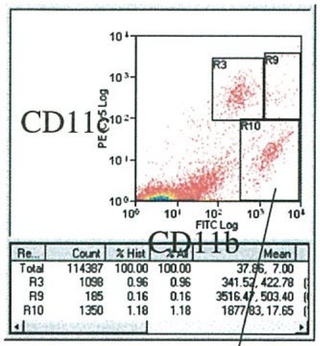


R3 = Inflammatory Macrophages (iMacs)
R9 = dendritic cells
R10 two poulations subdivide by Gr1
Gr1 low = resident macrophage
Gr1 high = granulocyte

Gr1

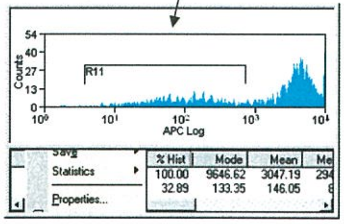


Start with ungated population

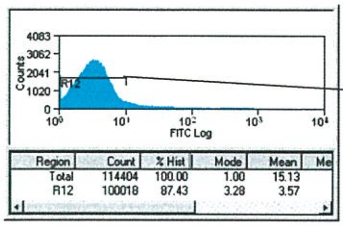


F4/80 a scavenger receptor on macrophages
Use F4/80 negative population since NKs are big and granulated like macrophages

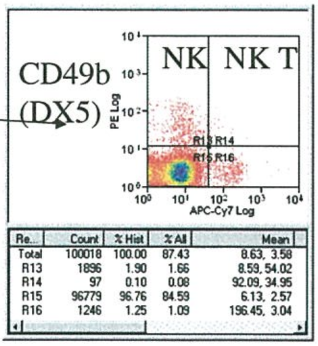


CD3 a T cell marker

Mice were euthanized and the pulmonary cavities were opened. After severing the descending aorta, the blood in the lungs was cleared by perfusion through the right heart with 5 ml of PBS containing 50 U of heparin (Sigma- Aldrich, St. Louis, MO) per ml until the lungs and liver became pale. Using an 18-gauge gavage needle, the trachea was cannulated, and 1 ml of PBS was slowly injected into the lungs and then withdrawn.

To obtain lung cell populations, the lungs were aseptically removed and minced. One lobes was saved and fixed in 1% paraformaldehyde/PBS for histology and immunohistochemistry. The remaining lobes were incubated in RPMI 1640 (Life Technologies, Gaithersburg, MD) containing collagenase XI (0.7 mg/ml; Sigma-Aldrich) and type IV bovine pancreatic DNase (30 mg/ml; Sigma-Aldrich) during 30–45 min at 37°C.

The action of the enzymes was stopped by adding 10 ml of RPMI medium containing 10% FBS, and digested lungs were further disrupted by gently pushing the tissue through a nylon screen (70 mm). The single-cell suspension was then washed and centrifuged at 200*g*. To lyse contaminating RBCs, the cell pellet was incubated during 30 seconds room temperature with 1.8 ml ddH2O, then mixed with 200ul 10X PBS.

*Intracellular staining for FoxP3*

Cells were surface stained as described, washed, and then fixedfor 5 min at 37° C in 4% paraformaldehyde. Cell suspensions were then permeabilized by incubating in PBS containing 0.1% Triton X-100 for 1 h on ice. Suspensions were washed and then incubated overnight with anti-FoxP3 Abs in PBS containing 0.5% BSA and analyzed the following day by flow cytometry.

[illegible]

Mϕ

CD4 - PE-Cy5 | CD11c PE-Cy5 | ✓
CD44 - FITC | CD206 biotin SA ~~[illegible]~~ PECy7 | FITC?
CD62L-APC | CD11b - ~~APC~~ FITC | APC
FoxP3 - PE | CD115 - PE | FITC
Gr1 ~~[illegible]~~ APC | ~~[illegible]~~ PECy7

4% PF in PBS
PBS + 0.1% Triton
PBS + 0.5% BSA
RPMI + Collagen/DNase

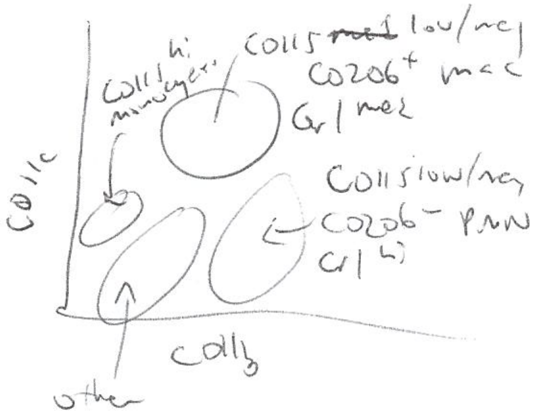

Sklm reanalysis 3/20/01

| | CD3+, CD8+, CD88 | CD8− other CD3+ | CD1d+ |
|---|---|---|---|
| 1 | .03 | | |
| 2 | .5 | | |
| 3 | .4 | | |
| 4 | .64 | | |
| 5 | .73 | | |
| 6 | .46 | | |
| 7 | .65 | | |
| 8 | .49 | | |
| 9 | .27 | | |
| 10 | .17 | | |

3/20/08 Reanalysis

| Autofluorescence Gate | | resident macs $11c^{hi}/11b^{lo}$ | PMN? monocyte/mac $11c^{lo}/11b^{hi}$ (Coll r*) | OC $11b^{e..}/11c^{hi}$ | CD206 |
|---|---|---|---|---|---|
| WT1 66543#1 | 1.1 | ~~46.5~~ 34 | ~~34~~ 46.5 | 2.5 | 0.5 |
| WT2 " #2 | 0.6 | ~~35~~ 36 | ~~36~~ 35 | 1.5 | 0.2 |
| WT3 " #3 | 3.6 | ~~35~~ 30 | ~~50~~ 35 | 4 | 5.3 |
| WT4 " #4 | 30 ??? | ~~57~~ 27 | ~~27.3~~ 57 | 3.2 | 13 |
| WT5 59548M tumor | 30 ?? | ~~24~~ 56 | ~~56~~ 24 | 2.17 | 20 |
| KO1 58848F wild | 8.6 | ~~16~~ 60.2 | ~~60.2~~ 16 | 5 | 4.5 |
| KO2 66546M wild | 1.8 | 32 | 37 | 1.8 | 0.16 |
| KO3 70087 F (moderate dz) | 0.9 | 80 | 3.5 | 2.3 | 0.35 |
| KO4 70087 M | 0.6 | 75 | 10 | 0.9 | 1.1 |
| KO5 58805 wild | 0.5 | 56 | 25 | 3.6 | 0.4 |

histology → relatively little to be seen
70087 M + F have a few early lesions
other three are healthy

7/20/08 Sukhu

APC+
PE+/-

| | $APC^{++}$, $PE^{++}$ | $\gamma\delta^-$, $O\chi\delta^-$, APC-$Cy_7^+$, $PECy_5^-$, $CD3^+$ | |
|---|---|---|---|
| 1 | 0 | 0.17 | .12 |
| 2 | 0.17 | 1.8 | 2.4 |
| 3 | .13 | 0.8 | 1.7 |
| 4 | 0.14 | 0.7 | 2.1 |
| 5 | 0.21 | 1 | 4 |
| 6 | .07 | 0.5 | 2.9 |
| 7 | .014 | 1.2 | 4.9 |
| 8 | .09 | 0.75 | 2.08 |
| 9 | 0.04 | 0.42 | 2.1 |
| 10 | .02 | 0.37 | 1.15 |

other cry pos for everything

NK skin re-analysis 3/20/08

- CD11 tetramer - APC
- γδ FITC
- CD3 APC-Cy7
- CD8 PECy5
- OX5 - PE

W/ lung

what do we see?

1. a population that is bright on all channels
   - FITC γδ
   - PECy5 CD8
   - APC CD3+ or CD1d+?
   - PE OX5

2. 2 purple populations
   - CD8 - PE-Cy5 · (−)
   - CD1d+ or − APC (+) + (−)
   - OX5 PE (−)
   - γδ (−)

3. ~~a CD8 dim~~ OX5+, CD1d−, CD3−, CD8−, γδ ~~[illegible]~~ + + (−)

   2 populations?

[illegible] Analysis — Macs/OCs

**LUNG** Gate first on auto fluorescent cells

| | ↓ | 11c hi/11b lo | 11c lo/neg/11b hi | count + | 11b+/11c+ | CD206+ |
|---|---|---|---|---|---|---|
| WT1 | 1.3 | 52 | 34 | 100% | 4 | 1 |
| WT2 | 1.4 | 62 | 23 | 100% | 4 | 1 |
| WT3 | 1.6 | 55 | 30 | " | 3.4 | 0.3 |
| WT4 | 2.6 | 64 | 19 | " | 2.8 | 1 |
| WT5 | 2.7 | 60 | 23 | " | 4.2 | 2 |
| KO1 | 17 | 87 | 1.4 | " | 2.8 | 10.4 |
| KO2 | 2 | 83 | 8.4 | " | 2.4 | 2 |
| KO3 | 2 | 48 | 30 | " | 3.4 | 5.5 |
| KO4 | 6.6 | 87 | 3 | | 4 | 3.2 |
| KO5 | 9.6 | 93 | 0.7 | | 0.7 | 5.3 |

Higher [illegible]

SKIN UNGATED DUE TO KERATINOCYTE AUTO FLUORESCENCE

↓ very prominent

| | | | | |
|---|---|---|---|---|
| WT1 | 3.6 | 1.6 | 3.5 | N/A |
| WT2 | 2.2 | 0.8 | 1.9 | ↓ |
| WT3 | 0.5 | 0.05 | 0.4 | |
| WT4 | 2.3 | 0.25 | 0.6 | |
| WT4 | 2.2 | 0.24 | 3.9 | |
| KO1 | 0.3 | 0.07 | 0.3 | |
| KO2 | 1 | 0.07 | 0.2 | |
| KO3 | 0.7 | 0.07 | 0.3 | |
| KO4 | 0.9 | 0.09 | 0.13 | |
| KO5 | 0.8 | 0.05 | 0.2 | |

6/14/08

skin DNFB day 3

| | γδ+, CD1− | γδ+ CD1+ | DX5+, CD1− | DX5+, CD1+ |
|---|---|---|---|---|
| WT1 | 10 | 1 | 13 | 15 |
| WT2 | 10 | 3 | 14 | 10 |
| WT3 | 6 | 1 | 7 | 2 |
| WT4 | 7 | 1 | 6 | 14 |
| WT5 | 9.5 | 3 | 11 | 12 |
| KO1 | 2 | 0 | 2 | 1 |
| KO2 | 1 | 0.5 | 1.4 | 3.7 |
| KO3 | 2 | 1 | 3 | 3 |
| KO4 | 2 | 1 | 2 | 8 |
| KO5 | 1.5 | 1.5 | 1.6 | 1 |

National® Brand 42-381 50 SHEETS EYE-EASE® · 5 SQUARES 42-382 100 SHEETS EYE-EASE® · 5 SQUARES 42-389 200 SHEETS EYE-EASE® · 5 SQUARES

88 analysis | ↓ used skin pd.plo 3/20/08 + ↙ used skin pd 2.plo 6/19/08

| 3/20/08 | CON3 | 88 | INKT | C08 | NK cos to | ~~NK~~ cos to |
|---|---|---|---|---|---|---|
| WT1 | n/d | ~~[illegible]~~ 3.7[illegible] | 0.02 | 0.13 | 0.19 | |
| WT2 | | 5.7 | 0.26 | 2.8 | 2 | |
| WT3 | | 4.3 | 0.06 | 0.85 | 1.4 | |
| WT4 | | 6.3 | 0.04 | 0.5 | 1.1 | |
| WT5 | | 10.6 | 0.1 | 1.1 | 2.3 | |
| KO1 | | 4 | 0.05 | 0.8 | 1.35 | |
| KO2 | | 5.3 | 0.14 | ~~[illegible]~~ 1.75 | 3.5 | |
| KO3 | | 4.9 | 0.09 | 0.6 | 2 | |
| KO4 | | 2.5 | 0.05 | 0.6 | 1.1 | |
| KO5 | | 2.2 | 0.04 | 0.25 | 1.1 | |

| 6/19/08 | | | | | | |
|---|---|---|---|---|---|---|
| WT1 | ~~n/d~~ 0.01 | 7 | 0.47 | ~~[illegible]~~ 1.6 | ~~[illegible]~~ 1.0 | ~~[illegible]~~ |
| WT2 | ~~[illegible]~~ 0.3 | 13 ~~[illegible]~~ | 0.10 | ~~[illegible]~~ 0.67 | ~~[illegible]~~ 1.5 | 0.9 |
| WT3 | 0.07 | ~~[illegible]~~ 4 | 0.05 | ~~[illegible]~~ 0.2 | 0.9 | ~~[illegible]~~ 0.[illegible] |
| WT4 | 0.3 | ~~[illegible]~~ 5.3 | 0.2[illegible] | 0.35 | ~~[illegible]~~ 1.5 | 0.4 |
| WT5 | ~~[illegible]~~ 0.3 | 8.5 | 0.10 | ~~[illegible]~~ 0.3 | ~~[illegible]~~ 1.4 | 0.[illegible] |
| KO1 | 0.1 | 2.6 | 0.01 | 0.03 | 0.2 | |
| KO2 | 0.2 | 1.3 | 0.03 | 0.16 | 0.2 | |
| KO3 | 0.1 | 2.5 | 0.05 | 0.08 | 0.5 | |
| KO4 | 0.4 | 1.5 | 0.05 | 0.14 | 0.16 | |
| KO5 | n/d | 2.9 | ~~0.0[illegible]~~ n/d | ~~0.01~~ n/d | 0.6 | |

6/20/08 CD4 FoxP3 Analysis

→ APC @ 900V but still too low

generally it appears FoxP3+ are also CD44+

| | CD4+ (of total) | CD4+, FoxP3+ (of total) | % of CD4+ |
|---|---|---|---|
| WT1 | ~~18~~ 15 | ~~1.8~~ 2.5 | 17 |
| WT2 | 18 | 1.8 | 10.4 |
| WT3 | 14.3 | 1.2 | 8.5 |
| WT4 | 20.3 | 1.4 | 6.9 |
| WT5 | 12.1 | 1.6 | 12.8 |
| KO1 | 6 | 1.8 | 31 |
| KO2 | 13 | 1.3 | 10 |
| KO3 | 9.3 | 0.7 | 7.4 |
| KO4 | 6.1 | 1.6 | 27 |
| KO5 | 5.6 | 0.7 | 13.2 |

8/14/08 digest 0.7mg/ml Collagenase XI + 0.7mg Type 2

| | x5 | x1/ml |
|---|---|---|
| W1L | 2e4 | 1e5 |
| W1R | 10e4 | |
| W2L | 2e4 | |
| W2R | 4e4 | |
| W3L | 4e4 | |
| W3R | 10e4 | |
| W4L | 1e4 | |
| W4R | 4e4 | |
| W5L | 6e4 | |
| W5R | 12e4 | |
| K1L | 1e4 | |
| K1R | 2e4 | |
| K2L | 5e4 | |
| K2R | 7e4 | |
| K3L | 2e4 | |
| K3R | 4e4 | |
| K4L | 2e4 | |
| K4R | 3e4 | |
| K5L | 1e4 | |
| K5R | 2e4 | |

10

L → NK/γδ only

R → hope we have enough to see 1e5/tube

A - CD4 Pacific Blue / CD44 FITC / CD62L APC

B CD8 " " / " " / " "

C γδ FITC / CD1d APC / OX5 PE / NKG2D PECy7

D CD11c Pacific Blue / CD11b FITC / Gr1 APC / CD117 PE

10 tubes except C = 20

1mg 2mg

12/22/08

| CD4+ T | CD8+ T | CD19+ B | NK/NK T | Monocyte/DC | Color |
|---|---|---|---|---|---|
| CD3 | CD3 | CD3 (-) | CD3 | | APC CY7 |
| | | | | | PE CY5 |
| CD4 | CD8 | CD19 | CD8 | CD11c | Pacific Blue |
| CD44 | CD44 | CD5 | γδ T cell | CD11b | FITC |
| CD62L | CD62L | CD62L | CD1d | GR1 | APC |
| | | | | CD206-biotin | PE Cy7 |
| FoxP3 | | IgD | DX5 (CD49b) | CD115 | PE |
| | | | | | |

NKG2D biotin next time

*Intracellular staining for FoxP3*

Cells were surface stained as described, washed, and then fixed for 5 min at 37° C in 4% paraformaldehyde. Cell suspensions were then permeabilized by incubating in PBS containing 0.1% Triton X-100 for 1 h on ice. Suspensions were washed and then incubated overnight with anti-FoxP3 Abs in PBS containing 0.5% BSA and analyzed the following day by flow cytometry.

[illegible]

16 samples

spin @ 1800 rpm

- 1% PF in PBS 15' RT
- WASH 1X PBS + BSA
- Permeabilize in 0.1% Saponin in PBS 30' RT
- O/N in 0.1% Saponin / PBS / BSA @ 4°C FoxP3

12/22/08

One lobe ↓

1e4

p02

| | | | skin | lung | | |
|---|---|---|---|---|---|---|
| | | | 5x10^4 | 10x10^4 | | |
| WT1 | | | 4 | 30 | 3e6 | 300 |
| WT2 | | | 14 | 200 | 2e7 | 50 |
| WT3 | | | 6 | 100 | 1e7 | 100 |
| WT4 | | | 4 | ~~[illegible]~~ [illegible] 80 | 8e6 | 125 |
| WT5 | | | 12 | ~~[illegible]~~ 50 | 5e6 | 200 |
| KO1 | 70087 | 97.7% ✓ | 3 | 80 | 8e6 | 125 |
| KO2 | 78640 | 90.1% ✓ | 6 | 80 | | |
| KO3 | 72790 | 85% ✓ | 5 | 120 | | |
| KO4 | 75875 | ~~[illegible]%~~ 70 % n3 | | 100 | | |
| KO5 | 74890 | ~~[illegible]%~~ 77% | 10 | 60 | | |

50 mL ✓

10 petri dishes ✓

20 tubes for flow 10 ear, 10 lung
+ 6 spleen
+ 4 blood

30 total ✓

30 flow Tubes

| | | 10x10^4 | |
|---|---|---|---|
| Spleen | WT1 | 33 | 3e6 |
| | WT2 | 90 | 9e6 |
| | WT3 | 90 | 9e6 |
| | KO1 | 70 | 7e6 |
| | KO2 | 120 | 1.2e7 |
| | KO3 | 40 | 7e6 |

- FoxP3 protocol from Tahun
- Collagenase for lungs + skin

15 tubes for KO mice

1. BAL
2. IHC
3. RNA

} mouse ID #, date, FKO

15 ml conical

(20) 50 mL conicals

ONFB combined

Ecr analysis II

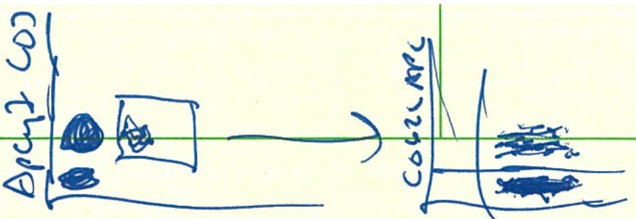

| | COM | A | B | C | D |
|---|---|---|---|---|---|
| W1 | 8.4 | 3 | 50 | 0 | 47 |
| W2 | 11.1 | 1 | 50 | 0.3 | 49 |
| W3 | 7.8 | 0.4 | 50 | 0 | 50 |
| W4 | 16.4 | 0.6 | 42 | 0.3 | 57 |
| W5 | 9.6 | 0.9 | 34 | 0.8 | 64 |
| K1 | 9 | 0.7 | 31 | 1.3 | 67 |
| K2 | 4.9 | 1.4 | 42 | 1.5 | 55 |
| K3 | 10.1 | 0.4 | 36 | 0.8 | 63 |
| K4 | 11 | 0.5 | 21 | 2.5 | 76 |
| | | | | | |
| W1 | 23 | 0 | 3 | 3 | 94 |
| W2 | 34 | 0.3 | 4 | 3 | 93 |
| W3 | 11 | 0.8 | 5 | 4 | 91 |
| W4 | 21 | 1.6 | 14 | 7 | 77 |
| W5 | 7.9 | 3.3 | 12 | 7.2 | 78 |
| W6 | 26.8 | 0 | 16 | 0.4 | 84 |
| K1 | 14 | 0.3 | 19 | 1.6 | 80 |
| K2 | 15 | 0.2 | 9 | 0.9 | 90 |
| K3 | 7.5 | 0.8 | 12 | 10 | 77 |
| K4 | 8.7 | 0.1 | 14 | 1.4 | 85 |
| K5 | 9.2 | 0.4 | 12 | 1.2 | 87 |
